# Machine learning based analysis of diesel engine performance using Fe₃O₄ nanoadditive in sterculia foetida biodiesel blend

**DOI:** 10.1038/s41598-025-26178-8

**Published:** 2025-11-07

**Authors:** Srinivasarao Mylapalli, Yaswanth Kumar Reddy Maddi, Joga Rao Bikkavolu, Battula Suryanarayana Murthy, Gandhi Pullagura, H. Ravi, Milon Selvam Dennison, Debabrata Barik

**Affiliations:** 1 Department of Mechanical Engineering, Sanketika Vidya Parishad Engineering College, Visakhapatnam, Andhra Pradesh India; 2 Department of Mechanical Engineering, Godavari Global University, Rajamahendravaram, 533296 India; 3 Department of Mechanical Engineering, GITAM (Deemed to be University), School of Technology, Visakhapatnam, 530045 India; 4 Department of Mechanical Engineering, GITAM (Deemed to be University), School of Technology, Hyderabad, 502329 India; 5 Department of Mechanical Engineering, Kampala International University, Western Campus, P.O. Box 71, Ishaka-Bushenyi, Uganda; 6 Department of Mechanical Engineering, Karpagam Academy of Higher Education, Coimbatore, 641021 India; 7 Centre for Energy and Environment, Karpagam Academy of Higher Education, Coimbatore, 641021 India

**Keywords:** Sterculia foetida biodiesel, Fe_3_O_4_ nanoparticles, Diesel engine, Performance, Emissions, Machine learning, Chemistry, Energy science and technology, Engineering, Environmental sciences, Materials science, Nanoscience and technology

## Abstract

This investigation examines the influence of Fe₃O₄ (magnetite) nano additions in sterculia foetida methyl ester (SME) mixtures on diesel engine performance, combustion, and emissions. SME was produced using transesterification and dispersed with surface modified Fe₃O₄ nanoparticles (NPs) employing probe-type ultrasonication to achieve uniform distribution. Engine tests were performed using pure diesel, a 25% SME blend-75% diesel (SME25), and Fe₃O₄ dispersed SME25 blends at concentrations of 50, 75, and 100 ppm. The results showed that the engine performance measures such as brake thermal efficiency (BTE) increased by 6.69% and specific fuel consumption (SFC) reduced by 7.23% for SME + 100Fe sample than SME25 mix. For the same blend, combustion metrics, such as cylinder pressure (CP) and heat release rate (HRR), increased by 4.46% and 24.21% respectively. Furthermore, at greater loads, the SME25 + 100Fe mix reduced carbon monoxide (CO), hydrocarbon (HC), nitrogen oxide (NOx), and smoke emissions by 23.92%, 22.42%, 5.38%, and 3.61%, respectively. A machine learning (ML) based computational model was created to predict engine performance and emission characteristics across various nano fuel blends. The model achieved great prediction accuracy, with correlation coefficients (R) ranging from 0.9973 to 0.99995 and Root Mean Squared Error (RMSE) and Mean Absolute Percentage Error (MAPE) values within acceptable bounds. The study confirms that Fe₃O₄ nano fuel blends improve engine efficiency, decrease emissions, and benefit from the integration of ML for accurate and data-efficient performance modelling. This technique is found to be potential for sustainable fuel technologies.

## Introduction

The global energy needs have been rising exponentially due to rapid industrialization, urbanization, and increased transportation needs. A significant portion of this energy is derived from fossil fuels, particularly diesel, which continues to power a vast number of internal combustion (IC) engines in sectors such as transportation, power generation, and agriculture. However, the extensive use of fossil fuels contributes to the depletion of non-renewable energy resources and has a detrimental impact on the environment due to greenhouse gas emissions and air pollution. These growing concerns have driven the search for alternative, sustainable, and eco-friendly fuels that can partially or fully replace conventional diesel^1,2^.

Biodiesel has gained a promising substitute to diesel fuel due to its renewable nature, biodegradability, and compatibility with existing Compression Ignition (CI) engines without major modifications. Among various biodiesel feedstocks, Sterculia foetida has attracted attention because of its high oil content, non-edible nature, and widespread availability in several regions. The crude oil cannot be used directly in engines due to operational issues like injector clogging and other engine malfunctions. Therefore, transesterification effectively converts raw oil into quality biodiesel with improving fuel properties compared to pyrolysis or emulsification methods. Biodiesel has drawbacks, including lower heating value, higher viscosity, and blend compatibility issues that may require engine and fuel modifications^3,4^.

Researchers have extensively studied fuel modification techniques, particularly the use of fuel additives such as oxygenated compounds and nanomaterials (NMs) in diesel/biodiesel blends. Among these, NMs have emerged as effective octane enhancers in CI engines. Nanofuels are prepared by combining NMs with base fuels such as diesel, biodiesel-diesel blends, biodiesel-diesel-water emulsions, and alcohol additives such as ethanol and butanol. Commonly employed nanomaterials include cerium dioxide (CeO₂), aluminium oxide (Al₂O₃), titanium oxide (TiO₂), copper oxide (CuO), zinc oxide (ZnO), iron oxides, and carbon nanotubes (CNTs). These additives have significantly improved engine performance parameters by efficient combustion and lowering hazardous pollutants^5,6^. Seela et al.^7^ found that adding 100 ppm of CeO₂ NPs to 20% mahua methyl ester increased BTE by 1.8% and decreased SFC by 1%. Additionally, CO and HC emissions fell by 6–8% and 6–9% respectively. In another investigation, Murgan et al.^8^ noticed that dispersing Graphene Oxide (GO) NPs at a dosage of 50 and 100 ppm into palm oil-based methyl ester enhanced engine performance by up to 0.9%, resulting in a 0.03 kg/kW-h reduction in fuel consumption and a considerable decrease in harmful emissions. Rangabashiam et al.^9^ studied TiO₂ NPs at different concentrations in 100% Pongamia biodiesel and discovered that 100 ppm blends decreased HC and smoke emissions by 2.1% and 2.7%, respectively, at maximum load, and a 3.8% drop in nitrogen. Kumar et al.^10^ experimented with Pongamia-based ferrofluid nano-included fuel blends on a Kirloskar TV1 engine, observing an 8% reduction in BSFC and lower CO and HC emissions correlated to conventional blends. Khatri et al.^11^ further demonstrated the efficacy of ZnO NPs in diesel fuel, with an optimal concentration of 20 mg leading to a 15.58% improvement in BTE and a substantial decrement in NOx, CO, HC, Carbon dioxide (CO₂), and particulate matter emissions. Despite the growing body of experimental research, comprehensive analysis and prediction of overall engine performance with nano-assisted fuel blends remain complex, time-consuming, and resource-intensive.

In this scenario, incorporating Artificial Intelligence (AI) and ML has significant potential in the automotive industry to accurately forecast engine performance. Unlike traditional simulation-based or numerical models, which are often time consuming and computationally intensive, ML techniques offer faster and more precise alternatives for performance prediction. Although the literature on ML applications in engine analysis is still emerging, recent studies have highlighted a growing interest among researchers and industry professionals. Krishnasamy et al.^12^ implemented a Random Forest Regressor, trained on 324 experimental observations, and achieved an R² score of 0.997 with an 85:15 training-testing split. The model successfully predicted engine performance, and Lagrangian optimization was applied to determine the optimal input conditions: 12.48 Nm torque, 8.29 L/min biogas flow, 72.8% methane, and 68.3 °C intake temperature, maximizing thermal efficiency, and minimizing emissions. Sanjeevannavar et al.^13^ compared multiple ML algorithms, such as XGBoost, Random Forest, Decision Tree, and Linear Regression, for engine performance prediction. XGBoost outperformed all the other models, achieving R² = 0.999, RMSE = 0.540, and MAE = 0.292, confirming its superior accuracy. Yang et al.^14^ evaluated the predictive capability of Artificial Neural Network (ANN), Support Vector Regression (SVR), and Random Forest for forecasting the Indicated Mean Effective Pressure (IMEP) using inputs such as spark timing, engine speed, and load. ANN and SVR demonstrated excellent performance, with R² values near unity and low RMSE, indicating a strong correlation with the actual engine data. Sahin et al.^15^ employed an ANN, SVM, and XGBoost to predict overall engine performance and emission characteristics. The ANN model performed best for predicting BTE and SFC, while the SVM outperformed the exhaust gas temperature prediction (R² = 0.981). For emission prediction, XGBoost showed the highest accuracy in estimating CO₂ and hydrocarbon levels. Zhang et al.^16^ used SVR to model spark-ignition engine behavior, achieving an R² value close to 1 and minimal RMSE, indicating a highly accurate prediction of engine performance and emissions. Hung et al.^17^ investigated a range of ML models which contains ANN, ANFIS, GRNN, RBFN, and SVR, for predicting CI engine’s performance and emissions parameters. SVR consistently delivered the most accurate predictions across all parameters. Sonawane et al.^18^ applied the TOPSIS decision-making technique integrated with ML to predict the performance of a CI engine with various fuel blends. Their model achieved an R² greater than 0.95 and an MAPE between 1% and 5%, affirming its reliability.

Most prior studies have focused on conventional biodiesel feedstocks such as Jatropha, Karanja, and Mahua, whereas non-edible and underutilized oils such as Sterculia foetida remain largely unexplored. Although nanoparticles such as Al₂O₃, TiO₂, ZnO, and Fe₃O₄ have shown promise in improving biodiesel performance, their combined effect with Sterculia foetida biodiesel has not been investigated, and machine learning has been scarcely applied to nano-assisted biodiesel blends. Therefore, the research gap lies in the lack of an integrated study that couples Sterculia foetida biodiesel, Fe₃O₄ nanoparticle enhancement, and predictive ML modeling, which is addressed in the present work.

### Research novelty and objectives

This study presents a novel experimental and machine learning-based framework for evaluating the performance, combustion, and emission behavior of a CI engine fueled with Sterculia foetida biodiesel dispersed with magnetite Fe₃O₄ NPs. The research novelty lies in three dimensions: first, the use of Sterculia foetida oil, a non-edible and underutilized biodiesel feedstock with distinct fatty acid composition; second, the synergistic role of Fe₃O₄ nanoparticles as combustion catalysts and oxygen buffers to improve efficiency and reduce emissions; and third, the integration of advanced machine learning techniques for predictive modeling of engine responses, thereby minimizing exhaustive experimental trials.

The main objectives of this study include evaluating the effect of Fe₃O₄ NPs on the physicochemical and combustion properties of Sterculia foetida biodiesel, quantifying performance and emission changes across different blend ratios, and validating machine learning models through comparative analysis with experimental data. This integrated approach not only provides a deeper understanding of nano assisted biodiesel combustion but also establishes a scalable methodology for optimizing alternative fuels using data-driven tools. Ultimately, this research contributes to the development of cleaner, more efficient diesel engine technologies, offering a promising pathway toward sustainable and intelligent fuel systems.

### Materials and methods

The non-edible fruit Sterculia foetida is gathered from Arakuveli, Visakhapatnam. The heart-shaped Sterculia foetida fruit has 15–20 seeds and becomes pale after plucking. It is viable and appealing for biodiesel production due to its 50–60% oil content. The present section describes the materials and procedures utilised, such as Fe_3_O_4_ NPs used as fuel additives, SME generation from Sterculia foetida oil by transesterification, the preparation of blended fuels, and engine testing.

### Biodiesel Preparation from Raw sterculia foetida oil by transesterification

Biodiesel was produced from raw Sterculia foetida oil through a well know transesterification process, a chemical process that involves the reaction of oil with methanol (CH₃OH) in the presence of potassium hydroxide (KOH) as a catalyst to break down the oil molecules into methyl esters (biodiesel) and glycerol.

**Fig. 1:**
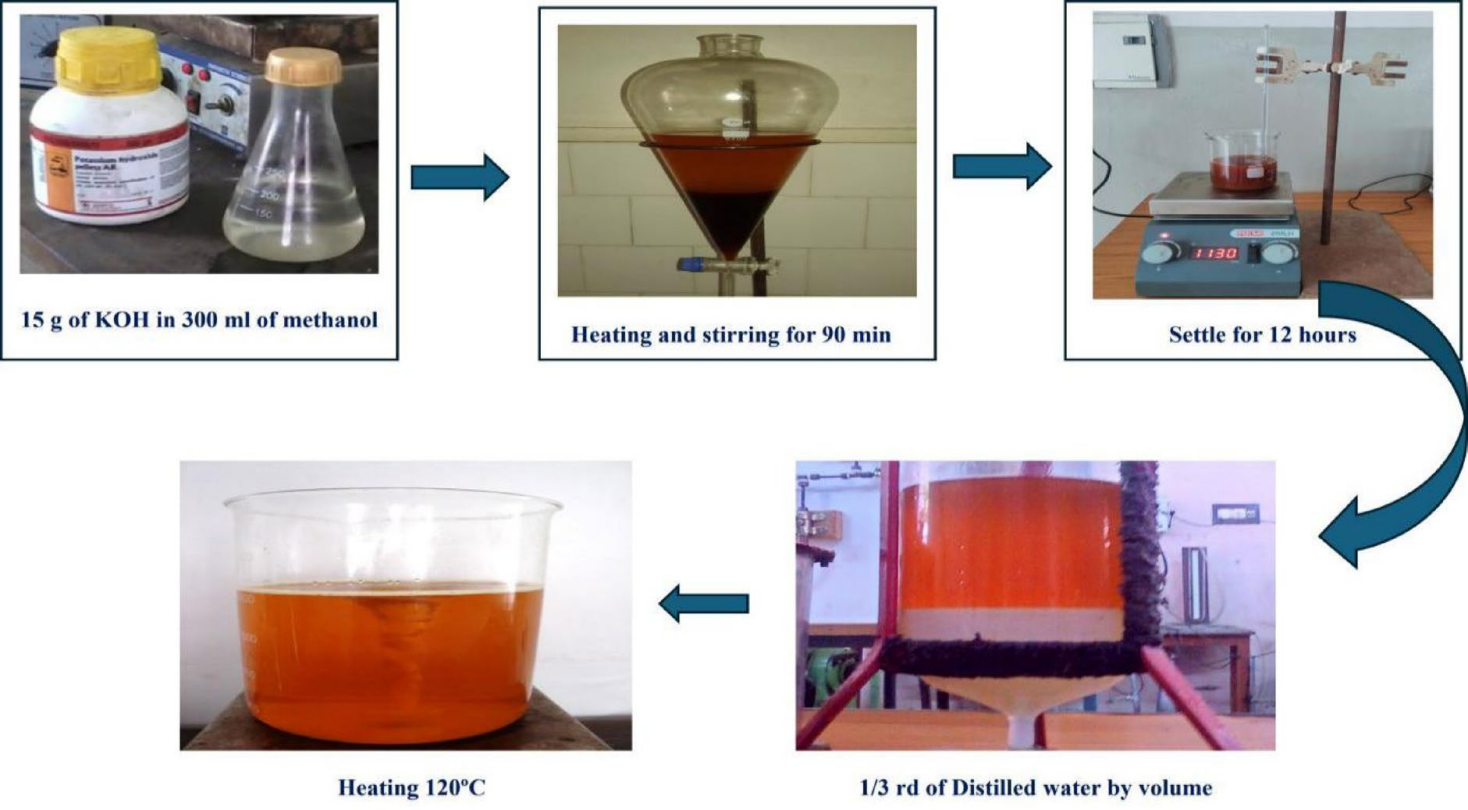
Illustration of the preparation of biodiesel using Sterculia foetida seed oil.

The method begins by diluting 15 g of KOH in 300 mL of methanol. Simultaneously, 1000 mL of raw Sterculia foetida oil was brought up to 60 °C with low-speed stirring. Once the oil had achieved the correct temperature, the KOH-methanol solution was added under airtight circumstances and agitated at 720 rpm for 2 h, maintaining the temperature at 60 °C. After this reaction, the solution was taken into a separating funnel and left to stabilize for 12 h. Biodiesel formed the upper layer, while glycerin settled at the bottom and was carefully removed. The biodiesel was then washed several times with warm distilled water to remove residual impurities until the wash water ran clear. Finally, the refined biodiesel was heated to 120 degrees Celsius to eliminate any leftover moisture before being stored for later use. This method effectively produces biodiesel with minimal impurities, making it suitable for engine applications. Figure 1. Shows the biodiesel preparation from seeds oil.

### Preparation of Fe_3_O_4_ based nanofuel

**Fig. 2:**
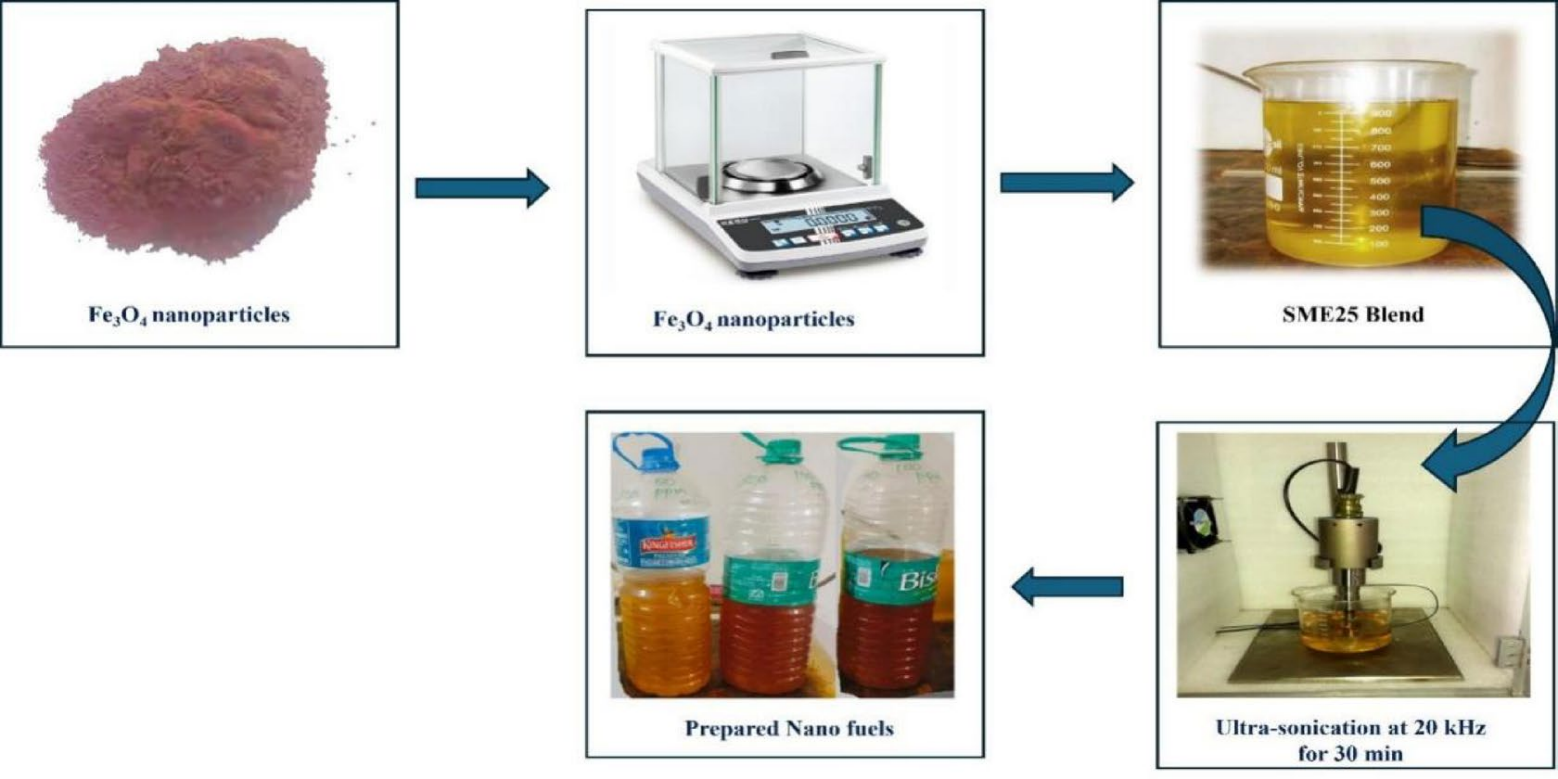
Preparation of nanofuel samples.

Figure 2 shows the method used to produce Fe_3_O_4_ based nanofuel. Table 1 shows the essential characteristics of NPs. The first sample, SME25, is made by combining 250 ml of SME with 750 L of diesel. Following that, Fe_3_O_4_ NPs were measured in needed amounts of 50, 75, and 100 ppm, and the NPs’ surface was changed with QPAN80 surfactant at a 1:1 to 1:5 ratio (NPs to surfactant) using a water bath and probe sonicator. The surface modification is found to be homogeneous at a ratio of 1:4, and the same ratio is used to modify the surface of the NPs. These surface modified Fe_3_O_4_ NPs at different concentrations (50, 75, and 100 mg/l) are mixed in a SME25 sample with the aid of a probe sonicator at a frequency of 20 kHz for 30 min to produce various nanofuel samples such as SME25 + 50Fe, SME25 + 75Fe, and SME25 + 100Fe. The prepared fuel samples are shown in Fig. 2. These samples are tested for physio-chemical properties, and the results are given in Table 2.

**Table 1:**
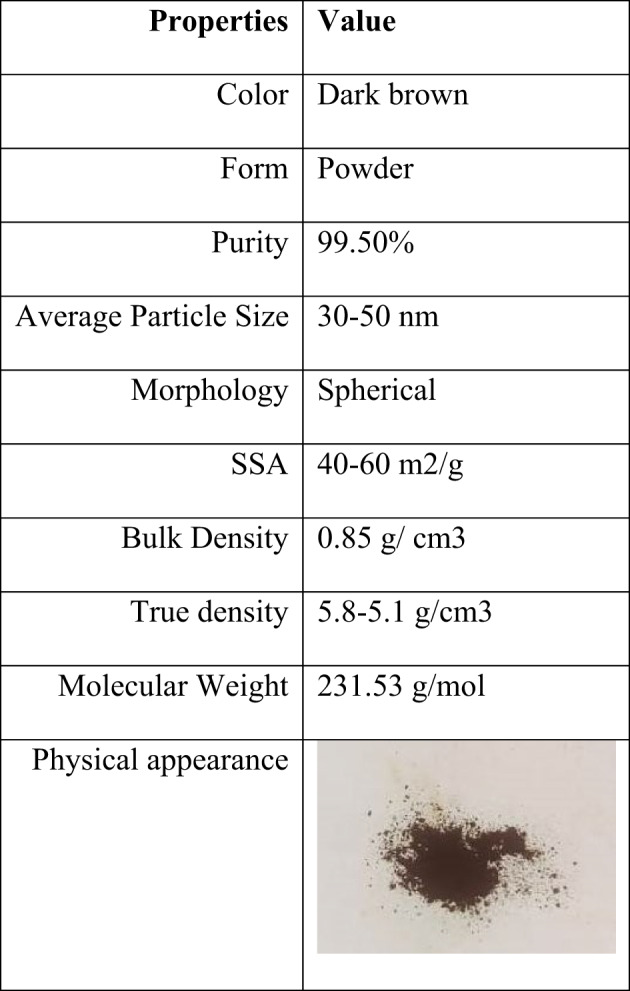
Properties of magnetite nanoscale particles.

**Table 2:** Presents the physio-chemical properties of the samples.

Fuel Characteristics	Testing method	Diesel Limit		Biodiesel Limit		Biodiesel from Sterculia foetida	SME25	SME25 + 50Fe	SME25 + 75Fe	SME25 + 100Fe
		ASTM D975	Diesel	ASTM D6751	EN14214					
Calorific value (MJ/kg)	ASTM D2015	42 to 46	42	-	35	37	38.5	39.5	40.4	40.8
Cetane Number	ASTM D976	≥ 47	56	47	-	57	57	58	58	59
Density (kg/m^3^ at 15 °C)	ASTM D1298	850	832	880	860 to 900	874	840	838	840	842
Kinematic viscosity (mm^2^/s @ 40 °C)	ASTM D445	2.0 to 4.5	3.2	1.9 to 6.0	3.5 to 5.0	4.5	4	3.7	3.75	3.8
Flashpoint (°C)	ASTM D93	60 to 80	48	100 to 170	120 min.	160	97	85	85	90
Fire point (°C)	ASTM D93	-	53	-	-	165	105	90	90	95
Cloud point (°C)	ASTM D2500	−35 to + 15	2	−3 to −12	-	3	2	−2	−2	−3
Pour point (°C)	ASTM D97	−15 to + 5	1	−15 to −16	-	−4	−4	−6	−6	−6

## Characterization of nanoparticles

**Fig. 3:**
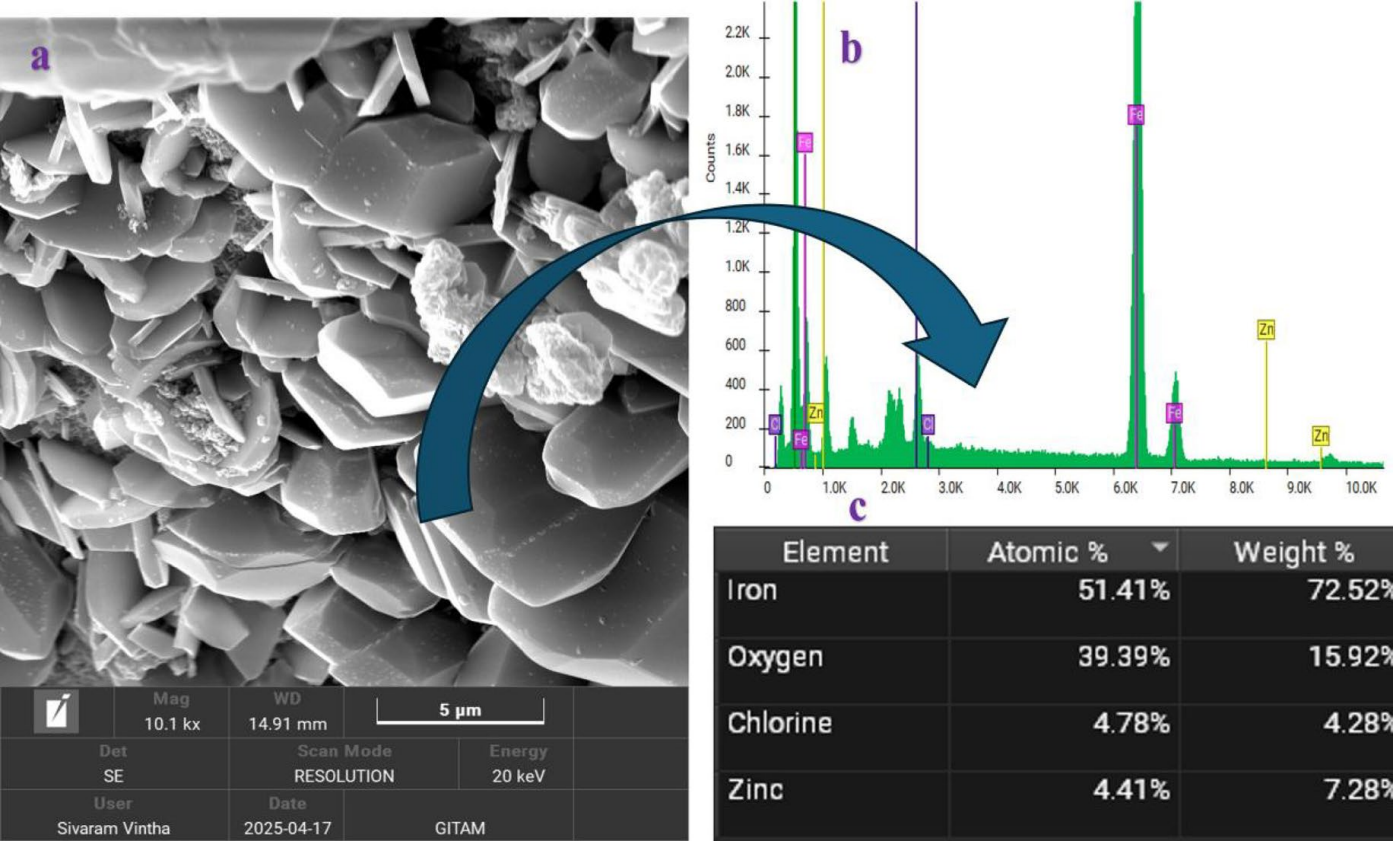
Represents the characterization of Fe_3_O_4_ **a**) FESEM, **b**) EDX **c**) Elements.

Fe_3_O_4_ NPs were obtained directly from the supplier “Platonic Nanotech.” To verify the existence of Fe_3_O_4_, Field Emission Scanning Electron Microscope (FESEM), and Energy Dispersive X-ray spectroscopy (EDX) studies were performed. The FESEM picture in Fig. 3(a) shows that Fe_3_O_4_ particles are grouped due to their smaller size. A Model FEI – Apreo LoVac FESEM was used for the analysis. FESEM image at magnification level 10.1 kX are shown in Fig. 3(a). Figure 3(b-c) depicts the EDX of Fe_3_O_4_, which displays the existence of chemical elements in a sample, with iron being disproportionately abundant.

## Experimental setup and test procedure

**Fig. 4:**
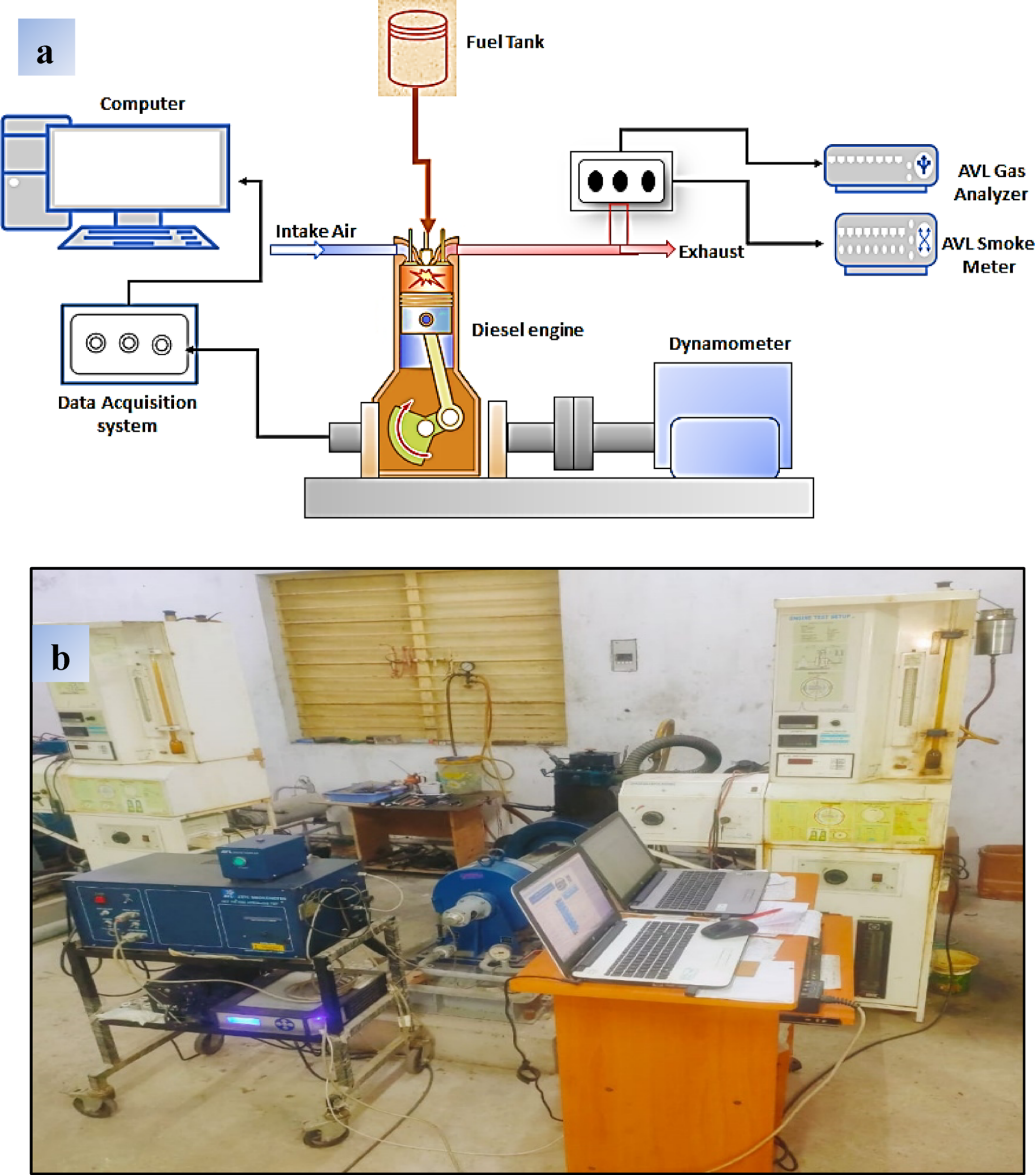
(**a**) and (**b**). Diagrammatic layouts and Pictorial representation of the Diesel engine.

The prepared nanofuels SME25 + 50Fe, SME25 + 75Fe, and SME25 + 100Fe, as well as the SME25 and diesel, were used on a four stroke, water cooled, mono cylinder, with a power output of 5.2 kW at 1500 rpm, operated at a rated speed of 1500 rpm under different engine loads (4.5, 8, 13.5, and 18 kg). A digital converter is connected to the computer once it has been coupled with all essential sensors and thoroughly tested to minimize errors. A computerized direct injection CI Engine with a preinstalled software called “engine soft” on a PC is used, and the data obtained is analyzed for 100 cycles. The schematic layout of the engine setup is depicted in Fig. 4(a), and the Pictorial representation of the CI engine is shown in Fig. 4(b). Table 3. Represents the engine setup details. The configuration consists of a 15 L diesel tank, a manometer, an air box, a fuel samples burette, a fuel pump, and a fuel nozzle (220 bar). A Yokogawa transmitter measures fuel flow, while a rotary encoder records speed. A temperature sensor (Type K, Type RTD, PT100), Air flow transmitter (Pressure transmitter, Range (-) 250 mm WC), a rotameter (Engine cooling 40‐400 LPH, Calorimeter 25‐250 LPH), Fuel flow transmitter, Load sensor (Load cell, type strain gauge, range 0‐50 Kg) and load indicator (Digital, Range 0‐50 Kg, Supply 230VAC) are used in the engine setup. Exhaust emissions (CO, HC, NOx, CO₂, O₂) are analyzed using an AVL Digas-444 five-gas analyzer, while smoke opacity is measured with an AVL 437 C smoke meter. Each test involved operating the engine at steady-state conditions for 10 min, followed by data recording.

**Table 3:** The details of the engine setup.

Engine Parameters	Specifications
Engine make	Kirloskar, TAF-1
No. of cylinders	1
No. of strokes	4
Dimensions (Bore and Stroke)	87.5 mm and 110 mm
Power Output	5.2 kW
Rated speed	1500 rpm
Compression ratio	17.5
Loading	Eddy current dynamometer
Gas analyzer and smoke meter	AVL Digas-444, and AVL437C
Ignition timing	23° bTDC

### Error analysis and uncertainties

Errors and uncertainties in testing can be caused by equipment, human observation, environmental conditions, or test preparation. Error analysis plays a vital role in ensuring the accuracy of test findings. Uncertainty evaluation is critical in complicated studies. Recognition and remedying frequent faults can improve the accuracy of experimental results. Repeated testing can help eliminate errors. To minimize mistakes, the tests are repeated five times. Table 4 shows how all performance variable faults are taken into account to establish the total uncertainty of the examination apparatus. The overall uncertainty of the experiment is calculated by Eq. 1.

The equation below evaluates the overall uncertainty:

1 $$\begin{aligned}& =\:\sqrt{{{(u}_{BTE})}^{2}\:+\:{\left({u}_{BSFC}\right)}^{2}\:+\:{\left({u}_{CO}\right)}^{2}+\:{\left({u}_{HC}\right)}^{2}+\:{\left({u}_{NOx}\right)}^{2}\:+{\left({u}_{\text{C}\text{O}2}\right)}^{2}+{\left({u}_{Smoke}\right)}^{2}} \\&= \sqrt{(1)^2 + (0.5)^2 + (1.5)^2 + (0.1)^2 + (1)^2 + (0.15)^2 + (1.5)^2}\\& \:=\:\pm\:\:2.60\%\end{aligned}$$

where:

- $\:{u}_{BTE}$: Uncertainty of Brake Thermal Efficiency.

- $\:{u}_{BSFC}$: Uncertainty of Specific fuel consumption.

- $\:{u}_{CO}$: Uncertainty of CO emission.

- $\:{u}_{HC}$: Uncertainty of HC emission.

- $\:{u}_{NOx}$: Uncertainty of NOx emission.

- $\:{u}_{\text{C}\text{O}2}$: Uncertainty of CO_2_ emission.

- $\:{u}_{Smoke}$: Uncertainty of Smoke.

**Table 4:** Accuracy and uncertainty of instruments.

Parameter	Range	Resolution	Uncertainty
Smoke Meter (Model: AVL 437 C)			
Smoke	0–100%	1%	± 1.5%
Exhaust Gas Analyzer (Model: AVL DI GAS 444 N)			
CO	0–15%	0.001% Vol	± 1.5%
HC	0–20000	10 ppm	± 0.1%
CO_2_	0–20%	0.1% Vol	± 0.15%
O_2_	0–25%	0.01% Vol	± 0.15%
NO_X_	0–6000	10 ppm	± 1%
AVL GH14d/AH1 Pressure transducer			
Pressure	0–100 bar	± 0.1 bar	± 0.2%
AVL 365 C Angle encoder			
Crank angle	0–720°	± 1%	± 0.1%
Data acquisition system			
Combustion characteristics	12 bit	± 0.01 bit	± 0.01%

### ML modeling

Machine learning (ML) modeling was employed to predict engine performance and emission parameters from the experimental dataset consisting of 20 observations and 4 variables (3 input features and 7 target variables). The data, representing various engine input conditions and corresponding responses, was imported into the Python environment using pandas along with supporting libraries such as numpy, matplotlib, and seaborn for preprocessing, analysis, and visualization. To improve model robustness, the dataset was partitioned using a training–testing split in combination with k-fold cross-validation, ensuring that prediction accuracy was not biased by a single data division.

Several regression-based algorithms, including Linear Regression and Random Forest Regressor, were applied to capture underlying relationships within the dataset. To ensure methodological rigor, hyperparameter tuning^19,20^ was performed using Grid Search optimization techniques for the Random Forest model. For the Random Forest model, hyperparameters such as the number of estimators, maximum depth, and minimum samples per split were systematically optimized. Model performance was evaluated using multiple metrics, including R², MAE, MSE, and RMSE. Among the tested algorithms, the tuned Random Forest model generally outperformed other approaches, demonstrating higher predictive accuracy and effectively capturing nonlinear dependencies in the data across most target variables. This refined ML framework thus provides a reliable and validated tool for predicting engine performance and emissions under varied operating conditions.

### Evaluation of performance parameters for the ML model employing statistical methods

Statistical measures, including regression coefficient (R^2^), Mean Relative Error (MRE), and RMSE, were investigated to determine the predictive power of the model strategies. The statistical metrics of the ML models are evaluated using Eqs. 2–4. The findings were utilized to evaluate the viability of machine learning in forecasting the efficacy of an IC engine, as well as the feasibility of fuel augmentation for the benefit of the environment.

2 $$\:{\text{R}}^{2}=1-\left\{\frac{\sum\:_{\text{i}=1}^{\text{n}}({{\text{E}}_{\text{i}}-{\text{M}\text{L}}_{\text{i}})}^{2}}{\sum\:_{\text{i}=1}^{\text{n}}({\text{M}\text{L})}^{2}}\right\}$$

3 $$\:\text{M}\text{R}\text{E}\:\left(\text{\%}\right)=\frac{1}{n}\sum\:_{i=1}^{n}\left|100\:\frac{{E}_{i}-{ML}_{i}}{{P}_{i}}\right|$$

4 $$\:\text{R}\text{M}\text{S}\text{E}\:=\sqrt{\frac{1}{n}\sum\:_{i=1}^{n}{({E}_{i}-{ML}_{i})}^{2}}$$

Where n = Number of data values, ML = Predicted values, and E = Test value.

## Results and discussions

### Brake thermal efficiency

**Fig. 5:**
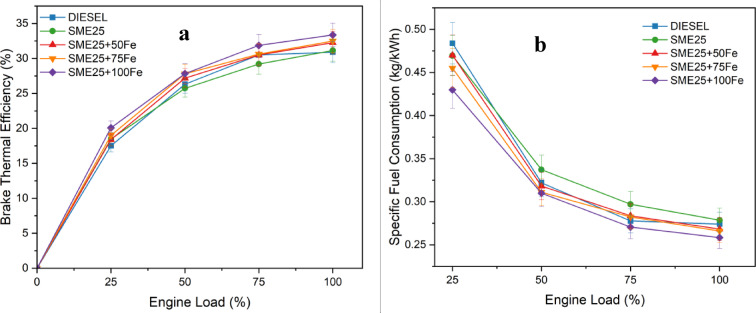
(**a**) Variation of BTE vs. Engine load, and (**b**). Variation of SFC vs. Engine load for various nano fluids blends.

Figure 5(a) presents the relationship between the BTE and engine load tested across a range of 0 to 18 kg. The BTE of the SME25 was lower than that of diesel, primarily because of its lower calorific value. However, the inclusion of Fe₃O₄ NPs resulted in significant improvements in the BTE, with increases of 3.45%, 4.19%, and 6.69% observed for 50, 75, and 100 ppm concentrations of Fe₃O₄ NPs, respectively. This improvement is primarily attributed to the oxygen-donating nature of Fe₃O₄ NPs, which facilitates the oxidative breakdown of carbon atoms, thereby providing more complete combustion. Additionally, NPs exhibit superior catalytic effect, improved fuel evaporation and atomization, shortened ignition delay, and reduced heat losses, collectively contributing to increased thermal efficiency^21^. Similar trends in the BTE improvement have been reported in previous studies involving nanoadditive-doped fuels^22^.

### Specific fuel consumption

Figure 5(b) shows the SFC for diesel, Sterculia foetida biodiesel blend (SME25), and SME25 with Fe₃O₄ NPs at varying engine loads. SME25 showed higher SFC than diesel across all loads due to its greater viscosity and density, affecting fuel evaporation and atomization. The addition of Fe₃O₄ NPs reduced SFC by 3.71%, 4.5%, and 7.23% at 50, 75, and 100 ppm concentrations, respectively. The addition of Fe₃O₄ NPs serves as a catalyst, enhancing combustion efficiency and reducing fuel consumption to achieve a similar engine power output. This effect can be ascribed to the creation of an oxygenated fuel blend when Fe₃O₄ is doped into the SME25, leading to a reduction in viscosity and density. These changes improve the air-fuel mixture and enhance atomization, resulting in better combustion characteristics. This trend of SFC reduction with Fe₃O₄ NPs additives has been similarly observed by Srinivasarao et al.^23^.

### Carbon monoxide

**Fig. 6:**
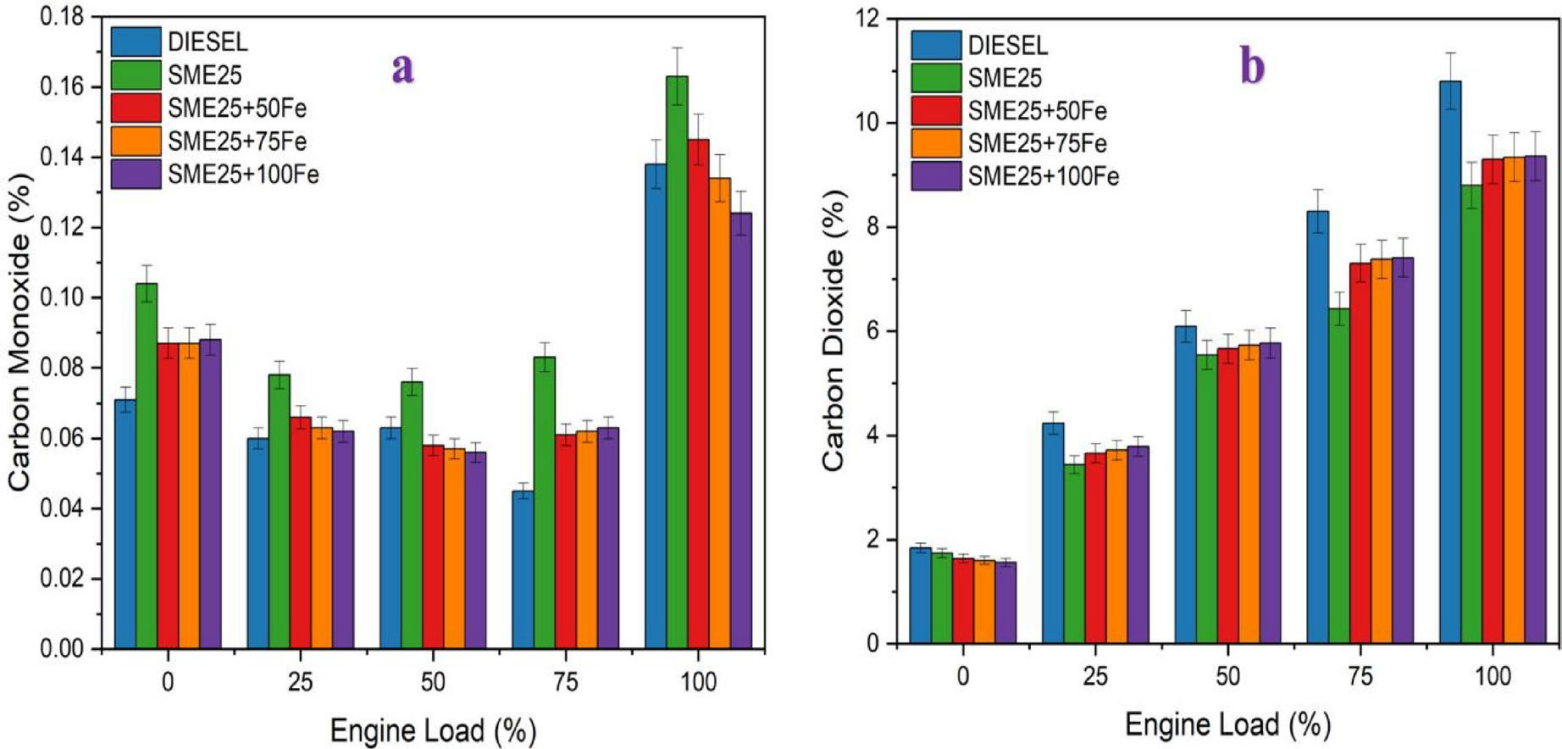
(**a**) Variation of CO vs. Engine load, and 6(**b**) Variation of CO_2_ against Engine load for various nano fluid blends.

Figure 6a illustrates the effect of CO emissions at different engine loads for the prepared fuel samples. The SME25 blend showed 15.33% higher CO emissions compared to diesel at higher loads, which is due to incomplete combustion resulting from its higher viscosity and lower volatility. However, the blend with Fe_3_O_4_ NPs of 50, 75, and 100 ppm fuel additive showed 11.04%, 17.79%, and 23.92% decreases in CO emissions, respectively. The inclusion of Fe_3_O_4_ NPs in SME25 decreased CO pollutants by dissolving into Fe and O atoms through the oxidation process. The O atoms increase the combustion properties, while the Fe atoms facilitate flame front movement between burnt and unburnt fuel particles. Hence, the combustion is complete. Ramakrishnan et al. (2019) noticed a similar reduction in CO gases using CNT NPs in neem oil-based biodiesel^24^.

### Carbon dioxide

Figure 6b illustrates the variation of carbon dioxide vs. engine load for different fuel blends. The SME25 sample decreased CO_2_ emissions by 18.51% than diesel at higher load. However, the addition of Fe_3_O_4_ NPs increased the CO_2_ emissions by 5.37%, 5.78%, and 5.98%, respectively, when correlated to the SME25 blend. This increase in CO_2_ is due to the NPs acting as combustion catalysts, promoting the complete oxidation of carbon-based fuel components. Tan et al.^25^ observed a similar increase in CO_2_ patterns in their research on bioethanol emulsions in biodiesel blends.

### Hydrocarbons

**Fig. 7:**
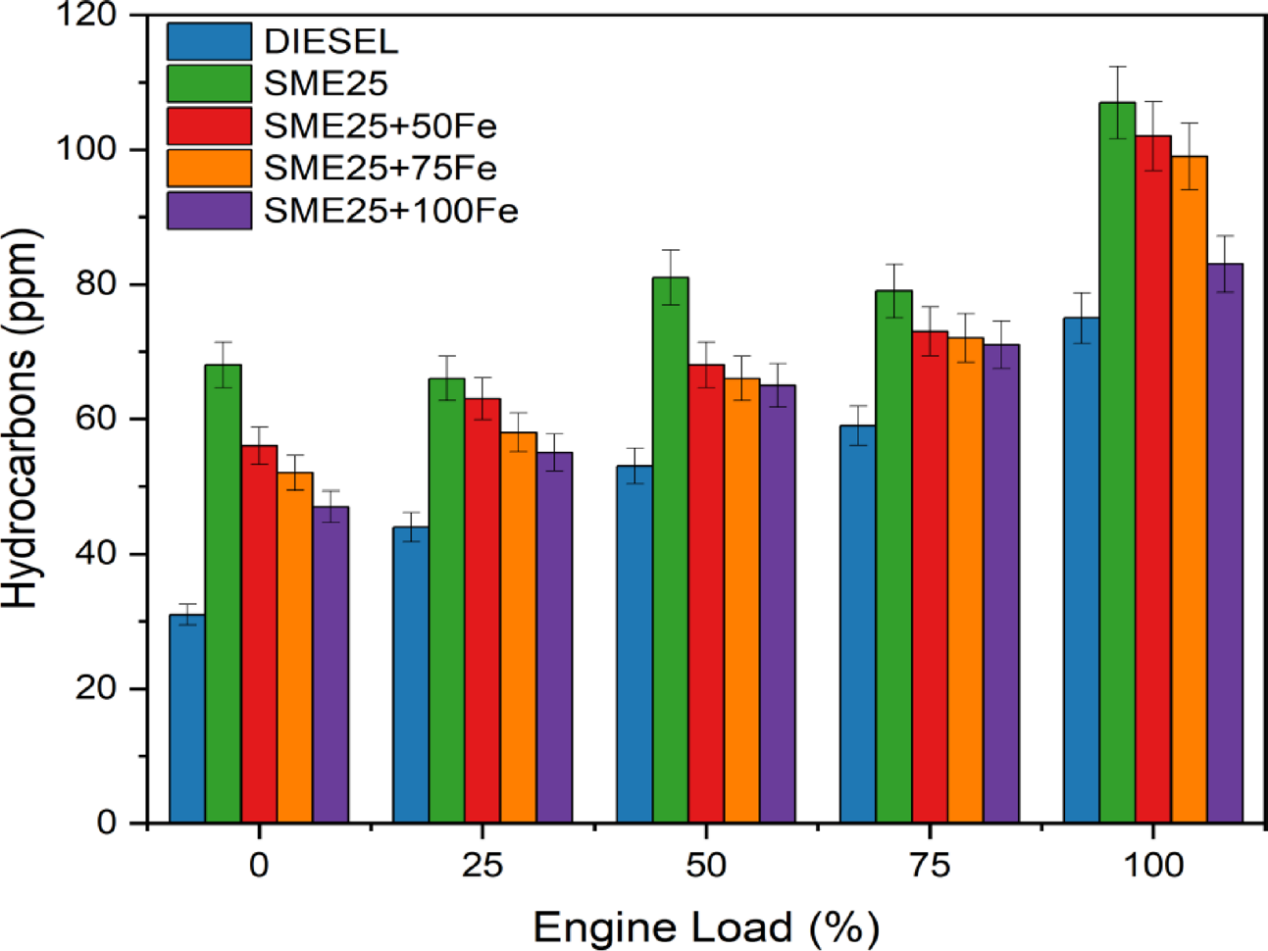
Variation of HC vs. Engine load for various nano fluids blends.

Figure 7 illustrates the variations in HC emissions at various engine loads for different samples. All fuel types had higher HC emissions as the load rose. This is because surplus fuel contributes to combustion without additional air, leading to a greater fuel-air mixture and increased HC emissions^26^. SME25 produces 29.90% more HC emissions than diesel at the highest level because of its greater viscosity, which promotes partial combustion and the formation of big droplets. Furthermore, SME25 includes unsaturated hydrocarbons, which lead to increased HC emissions that were lowered by 4.67%, 7.47%, and 22.42% with 50, 75, and 100ppm Fe_3_O_4_ NPs dosage respectively as in contrast to SME25. During burning, the NPs discharge oxygen molecules and have a large reactive surface, which aids in the oxidation of unburned fuel components and increases evaporation rate. This led to decrease rich mixture areas in the clearance volume and decreased HC pollutants. Hosseini et al.^27^ observed a similar decrease in HC for waste oil based domiciled biodiesel using nanoscaled CNTs NPs at various dosage levels.

### Nitrogen oxides

**Fig. 8:**
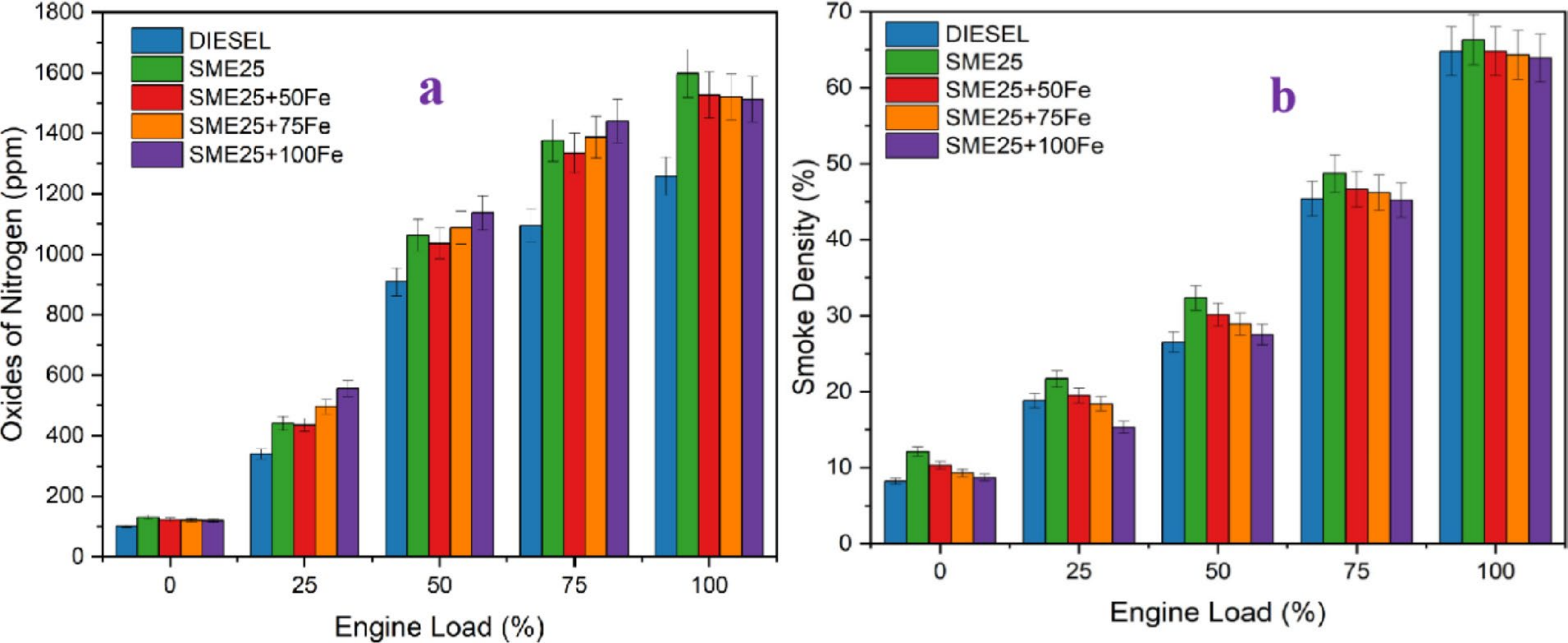
(**a**) Variation of NOx vs. Engine load, and (**b**) Variation of Smoke vs. Engine load for various nanofluid blends.

Figure 8a presents the effect of NOx emissions vs. engine loads for various prepared fuel samples. It is observed from the figure that the NOx emissions trend increases as the load increases. The SME25 blend exhibited 21.27% greater NOx emissions than diesel at higher loads. However, the addition of Fe_3_O_4_ NPs decreased NOx emissions by 4.50%, 4.88%, and 5.38%, respectively, than the SME25 blend. The high thermal conductivity of Fe₃O₄ NPs enhances the heat transfer within the combustion chamber, leading to lower peak combustion temperatures, which in turn reduces NOx formation. A similar investigation proved that the NOx emissions increase with ZnO NPs in Calophyllum biodiesel-diesel-Propanol blends as reported by Srinivasarao et al.^28^.

### Smoke density

Figure 8b depicts the variation of smoke on the engine loads of different fuels. The SME25 blend exhibited 2.26% higher smoke compared to diesel at higher load conditions. However, the addition of Fe_3_O_4_ NPs reduced smoke by 2.26%, 3.01%, and 3.61%, respectively, for 50, 75, and 100 ppm dosages when correlated to the SME25 blend. This decrease in smoke is due to the NPs improving atomization and fuel-air mixing, leading to better flame propagation and complete oxidation of carbon particles, thereby reducing smoke formation. Seela et al.^7^ reported similar smoke results using cerium oxide in mahua biodiesel blends.

### Cylinder pressure

**Fig. 9:**
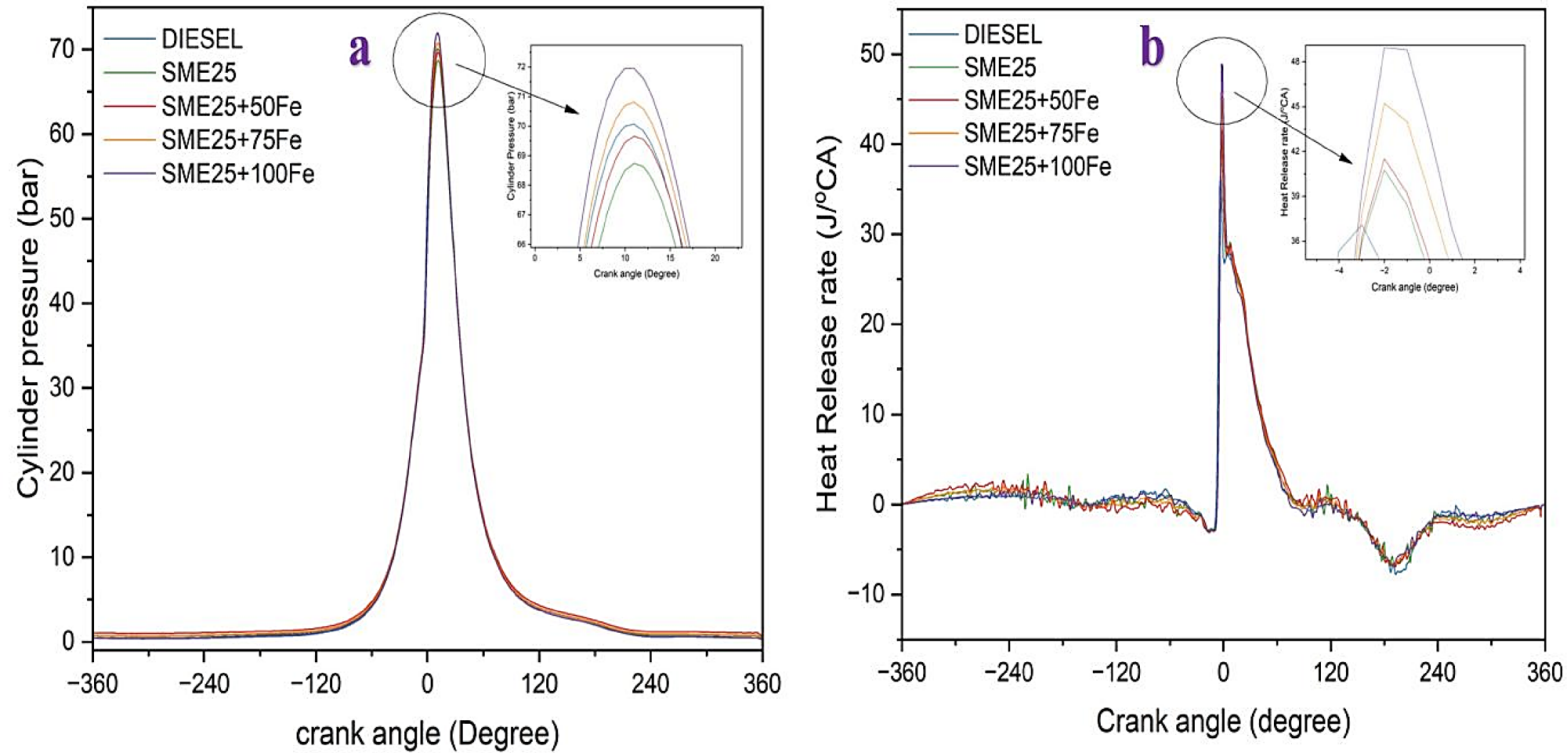
(**a**) Variation of CP vs. Crank angle, and (**b**) Variation of HRR against Crank angle for various nanofluid blends.

Figure 9a illustrates the variation of CP vs. crank angle for various fuel samples under higher load. The SME25 blend lowered the in-cylinder pressure by 1.89% compared with diesel. However, the addition of Fe_3_O_4_ NPs increased the cylinder pressure by 1.32%, 2.92%, and 4.46%, respectively, compared with SME25. NPs, which have a high surface-to-volume ratio, exhibit a catalytic effect and can also serve as oxygen buffers^29^. Hence, the combustion process is expedited. Similar findings for an increase in-cylinder pressure were reported by Bikkavolu et al.^30^ using carbon nanotube nanoadditives in yellow oleander biodiesel blends.

### Heat release rate

Figure 9b depicts the effect of HRR with the crank angle for various fuel mixes. The HRR of the SME25 mix was 8.98% less than that of diesel at full load because biodiesel has a shorter ignition delay, which is owing to its greater cetane number. Further, the surface tension and atomization of biodiesel result in a lower HRR. However, additions of 50, 75, and 100 ppm Fe_3_O_4_ NPs raised the HRR by 10.66%, 17.99%, and 24.21%, respectively, when contrasted with SME25. This HRR enhancement is ascribed to high-momentum liquid droplets and the catalytic activity of Fe_3_O_4_ NPs, which boosts the combustion phenomenon, allowing for comprehensive burning than SME25^31^. Nair et al.^32^ discovered a similar growing tendency when Al_2_O_3_ NPs were dispersed in waste plastic oil based biodiesel. The performance and emission characteristics are compared with the SME25 sample, and the same are given in Table 5.

**Table 5:** Comparison of performance and emission parameters of prepared samples when compared with the SME25 blend.

Fuel Blend	BTE (%)	BSFC (kg/kWh)	CO Reduction (%)	CO_2_ Reduction (%)	HC Reduction (%)	NOx Change (%)	Smoke Reduction
SME25 + 50Fe	↑3.45%	↓3.71%	↓11.04%	↑5.37%	↓4.67%	↓4.50%	↓2.26%,
SME25 + 75Fe	↑4.19%	↓4.5%	↓17.79%	↑5.78%	↓7.47%	↓4.88%	↓3.01%,
SME25 + 100Fe	↑6.69%	↓7.23%	↓23.92%	↑5.98%	↓22.42%	↓5.38%	↓3.61%,

## Application of machine learning modeling to experimental data

ML models were developed and evaluated using a Jupyter Notebook to analyze the performance and emission characteristics of Sterculia foetida biodiesel blends combined with Fe₃O₄ NPS fuel additives in a compression ignition engine. The models were trained using experimental datasets and assessed using various statistical performance metrics, demonstrating a high predictive accuracy across all key engine parameters^33,34^. The analysis workflow included data pre-processing, feature scaling, model training, and evaluation using regression metrics such as the R², RMSE, and MAPE^35^.

**Fig. 10:**
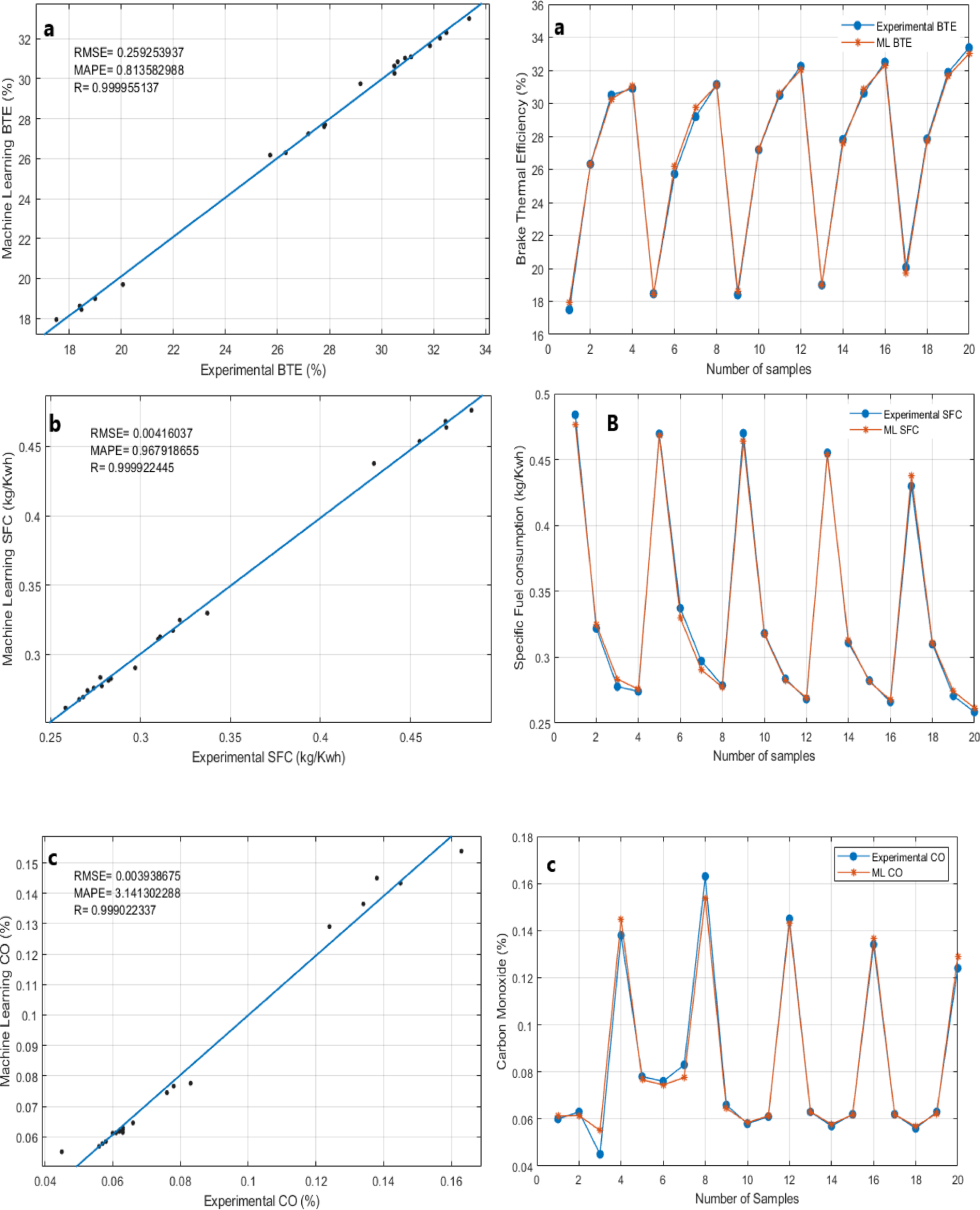

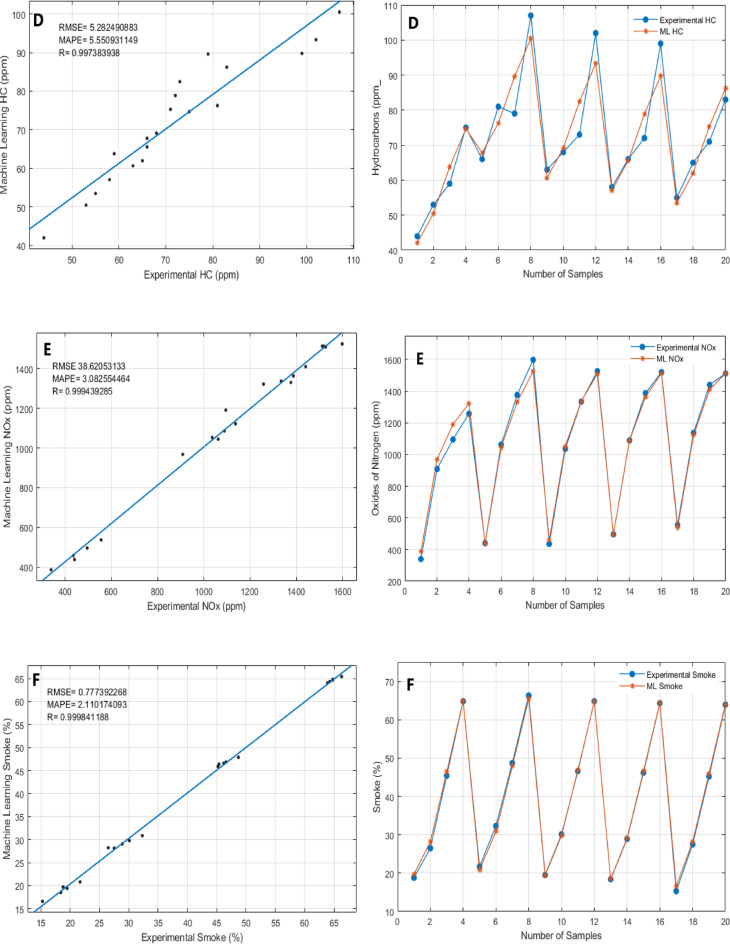

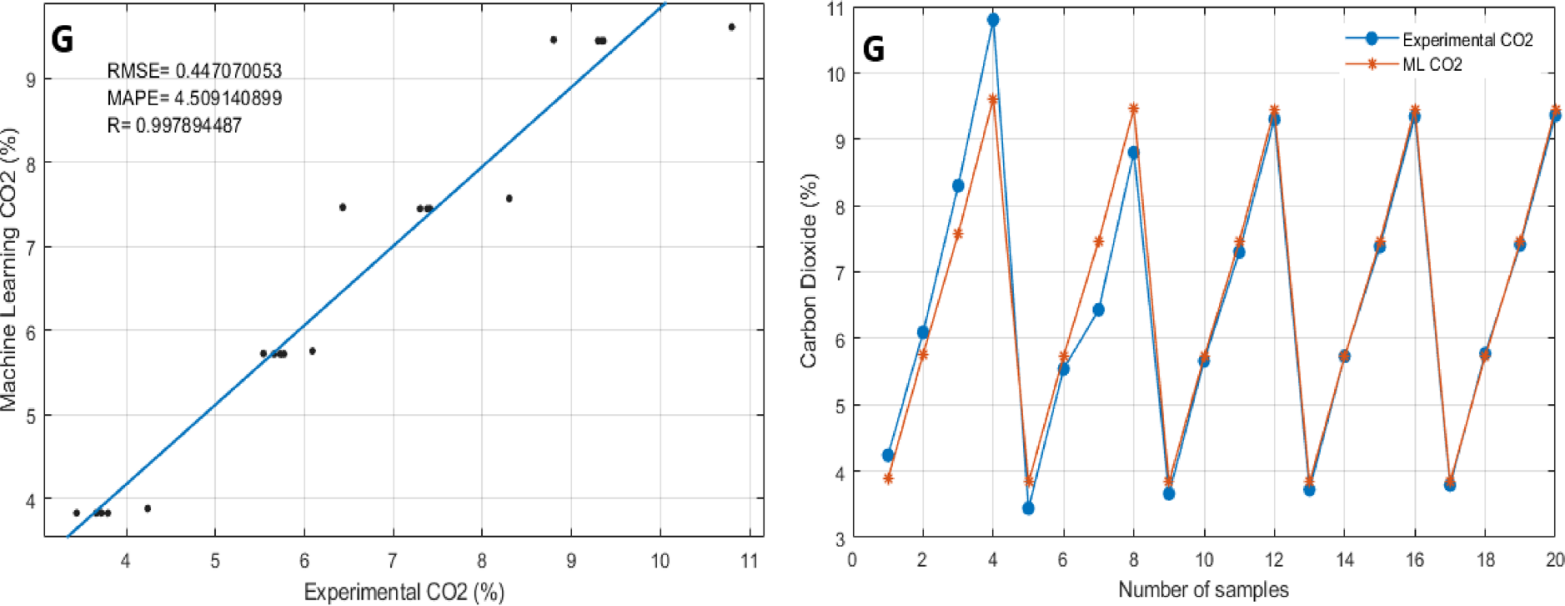
(a-g). Comparison of ML predicted and experimental results: (**a**) Brake Thermal Efficiency, (**b**) Specific Fuel Consumption, (**c**) Carbon Monoxide emissions, (**d**) Hydrocarbon emissions, (**e**) Nitrogen Oxides emissions, (**f**) Smoke opacity, (**g**) Carbon dioxide emissions.

Table 6 presents the statistical performance metrics of the Random Forest Regressor (RFR) model in predicting engine performance and emissions parameters, while the corresponding graphical comparisons are illustrated in Fig. 10(a–g). The Random Forest Regressor models demonstrated excellent predictive capability for both Brake Thermal Efficiency (BTE) and Specific Fuel Consumption (SFC), achieving R values of 0.99995 and 0.99992, RMSE values of 0.25925 and 0.00416, and MAPE values of 0.81358 and 0.69791, respectively (Fig. 10a and b). Similarly for emissions parameters like CO, HC, CO_2_, NOx and Smoke the R values are 0.99902, 0.99738, 0.99789, 0.999439, and 0.999841 and the RMSE values are 0.003938, 5.2824908, 0.44707, 38.620531 and 0.777392 and MAPE values are 3.141302, 5.5509311, 4.5091408, 3.082554 and 2.110174 respectively (Fig. 10c, d, e and f, & 10g). Thus, it can be concluded that Random Forest Regressor models performed well in performance and emissions parameters.

In terms of the comparison between test values and corresponding ML Predicted values obtained by the Random Forest Regressor model, are shown in Figs. 10 (a-g). The figures present ML prediction values and Test values. In all scenarios, the predicted values tend to be close to the test values. This indicates a good agreement between the ML prediction values obtained by the developed models and the test values. This finding also indicates that the Random Forest Regressor provides accurate predictive results.

Based on the results, it can be inferred that the Random Forest Regressor model can be used as a suitable tool in the prediction of performance and exhaust emissions of a diesel engine fuelled with Sterculia foetida biodiesel blends with Fe_3_O_4_ NPs. Moreover, all the obtained results of the developed models are highly correlated and precise. Overall, the Jupyter Notebook-based ML analysis proved to be an effective tool for modeling and forecasting overall engine performance parameters and emission characteristics. This approach offers a practical pathway for optimizing biodiesel fuel formulations and advancing sustainable combustion technologies through data-driven insights.

**Table 6:** Comparison between ML-predicted and test values for engine parameters.

Parameters	RMSE	MAPE	*R*
BTE	0.259253937	0.813582988	0.999955137
SFC	0.00416037	0.967918655	0.999922445
CO	0.003938675	3.141302288	0.999022337
HC	5.282490883	5.550931149	0.997383938
CO_2_	0.447070053	4.509140899	0.997894487
NOx	38.62053133	3.082554464	0.999439285
Smoke	0.777392268	2.110174093	0.999841188

### Cost–benefit analysis

A cost–benefit analysis was conducted to evaluate the economic viability of producing Sterculia foetida–based biodiesel enriched with Fe₃O₄ nanoparticles. The breakdown of production costs is presented in Table 7. The major contributors to the cost are seed procurement, oil extraction, and transesterification, while the additional expenses of nanoparticle synthesis and dispersion are relatively minor.

The total production cost of the biodiesel blend is estimated at ₹56–65 per liter, which is substantially lower than the prevailing market price of conventional diesel (₹90–96 per liter). This indicates a potential 30–35% cost advantage, demonstrating the techno-economic feasibility of using Sterculia foetida biodiesel as a sustainable fuel alternative.

**Table 7:** Cost Estimation of sterculia foetida biodiesel Production.

Materials	Estimated Cost (₹)
Raw seeds (per kg)	18–22
Oil extraction (per litre of oil)	20–25
Transesterification (per litre of biodiesel)	10–12
Fe₃O₄ nanoparticle procurement (per litre blend)	5 − 8
Blending & dispersion (per litre blend)	3 − 4

### Scalability and industrial relevance

Sterculia foetida oil, being a non-edible and regionally available feedstock, offers strong potential for large-scale biodiesel production without competing with food resources. The addition of Fe₃O₄ nanoparticles in biodiesel blend can be achieved through a sonicator, which is already an established technology. These nano-biodiesel blends can be utilized in conventional compression ignition (CI) engines with minimal modifications, thereby ensuring practical feasibility for field applications. However, challenges remain in achieving long-term dispersion stability, standardization of nanoparticle synthesis, and optimization of production costs for sustainable deployment.

The nano-biodiesel blends evaluated in this study demonstrated reduced emissions and improved thermal efficiency, making them compatible with stringent emission regulations. Their adaptability positions them as promising drop-in fuels for applications in transportation, agriculture, and decentralized power generation. Localized production and utilization can further enhance energy security, rural employment opportunities, and the growth of green industry initiatives. Nonetheless, successful industrial adoption will depend on achieving a favorable cost–benefit balance, regulatory approvals, and long-term validation of engine durability when operated with nano-biodiesel fuels.

## Conclusions

This research comprehensively explored the impact of Fe₃O₄ (magnetite) NPs as fuel additives in Sterculia foetida biodiesel blends on the performance, emission, and combustion behavior of a diesel engine. Additionally, the study evaluated the feasibility of employing ML models to accurately predict engine response parameters. The key conclusions drawn from the experimental and predictive analysis are as follows:

- The addition of Fe₃O₄ NPs to the Sterculia foetida biodiesel blend resulted in an increase in BTE by 3.45%, 4.19%, and 6.69%, and a corresponding reduction in SFC by 3.71%, 4.5%, and 7.23% for 50 ppm, 75 ppm, and 100 ppm NP concentrations, respectively.
- Emissions of CO decreased by 11.04%, 17.79%, and 23.92%, while HC emissions were lowered by 4.67%, 7.47%, and 22.42% with increasing NP concentrations.
- The Fe₃O₄ NPs blended nanofuels results in a reduction of NOₓ emissions by 4.50%, 4.88%, and 5.38%, and a decrease in smoke opacity by 2.26%, 3.01%, and 3.61% for 50 ppm, 75 ppm, and 100 ppm concentrations, respectively. Conversely, CO₂ emissions increased slightly by 5.37%, 5.78%, and 5.98%, indicating more complete combustion due to the catalytic activity of the NPs.
- The maximum improvement of in-cylinder pressure and HRR are 4.46%, and 24.21% for 100 ppm nanofuel indicate more efficient and intensified combustion facilitated by the presence of the NPs.
- The ML accurately predicted the output parameters, as indicated by high R^2^ values of BTE and SFC, having 0.99992 and emission-related predictions for CO, HC, CO₂, NOₓ, and smoke are consistently above 0.9973, with lower RMSE and MAPE values for both performance and emissions.

The main finding of this study is that the incorporation of Fe₃O₄ nanoparticles into Sterculia foetida biodiesel blends significantly enhances engine performance and combustion while reducing harmful emissions and that Random Forest modeling provides a reliable tool for accurately predicting these outcomes.

### Future scope

The investigation could be carried out with Sterculia foetida biodiesel-diesel blends with nano additives inclusion and tested in multi cylinder and turbocharged CRDI diesel engines. This study can be extended using machine learning modeling by comparing RF and deep learning and hybrid AI approaches for real time predictive optimization.

## Data Availability

The data will be made available upon request to the corresponding authors.

## Abbreviations

AI: Artificial Intelligence
Al_2_O_3_: Aluminium Oxide
ANN: Artificial Neural Network
BTE: Brake Thermal Efficiency
CeO_2_: Cerium Dioxide
CI: Compression Ignition
CNTs: Carbon Nanotubes
CO: Carbonmonoxide
CO_2_: Carbon Dioxide
CP: Cylinder Pressure
CuO: Copper Oxide
EDX: Energy Dispersive X-ray spectroscopy
FESEM: Field Emission Scanning Electron Microscope
GO: Graphene Oxide
HC: Hydrocarbon
HRR: Heat Release Rate
IC: Internal Combustion
IMEP: Indicated Mean Effective Pressure
MAPE: Mean Absolute Percentage Error
ML: Machine Learning
MRE: Mean Relative Error
NMs: Nano Materials
NOx: Nitrogen Oxide
NPs: Nanoparticles
R: correlation coefficients
R^2^: Regression Coefficient
RF: Random Forest
RMSE: Root Mean Squared Error
SFC: Specific Fuel Consumption
SME: Sterculia Foetida Methyl Ester
SME25: 25% SME blend-75% diesel
SVR: Support Vector Regression
TiO_2_: Titanium Oxide
ZnO: Zinc Oxide
